# Ethyl 10-cyano-7-hy­droxy-6-oxo-3-phenyl-8,9,10,10a-tetra­hydro-6*H*-benzo[*c*]chromene-10-carboxyl­ate

**DOI:** 10.1107/S2414314622001997

**Published:** 2022-02-25

**Authors:** H. Surya Prakash Rao, Prabakaran M, Jayaraman Muthukumaran

**Affiliations:** a Department of Chemistry, Pondicherry University, Puducherry 605014, India; b Vasista Pharma Chem Pvt Ltd, Gajularamaram, Hyderabad 500090, India; c Department of Biotechnology, School of Engineering and Technology, Sharda, University, Greater Noida 201306, India; University of Aberdeen, Scotland

**Keywords:** dibenzo­pyran, C—H⋯π inter­action, C—H⋯O inter­action, crystal structure

## Abstract

In the title compound, C_23_H_19_NO_5_, the cyano group adopts an axial orientation and the ester group an equatorial orientation. The dihedral angle between the pendant phenyl group and the benzene ring of the fused-ring system is 25.97 (8)°. Intra­molecular O—H⋯O and C—H⋯O hydrogen bonds are observed and the packing is consolidated by C—H⋯O and C—H⋯π inter­actions.

## Abstract 2

The packing of the title compound is consolidated by C—H⋯O inter­actions.

## Graphical abstract

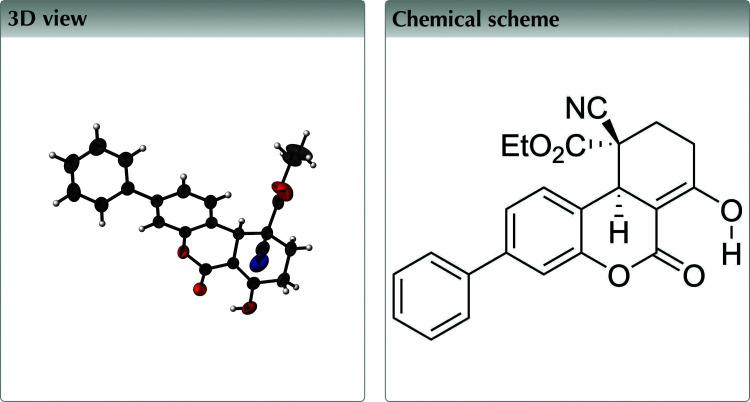

## Structure description

Dibenzo­pyran-6-ones (also called 6*H*-benzo[*c*]chromen-6-ones or 3,4,5,6-dibenzo-α-pyran­ones) form an important group of biologically active natural products that occur in bacteria, fungi, lichens, higher plants and animal waste (Bialonska *et al.*, 2009 ▸). Elsamitrucin, a dibenzo­pyran-6-one derived drug, is an efficient topoisomerase II inhibitor (Fiocchi *et al.*, 2011 ▸). As well as their biological activities, some dibenzo­pyran-6-ones have served as inter­mediates in the synthesis of more complex organic compounds (*see*, for example, Coghlan *et al.*, 2001 ▸). As a part of our ongoing studies in this area, we now describe the synthesis and crystal structure of the title compound.

**Figure 1:**
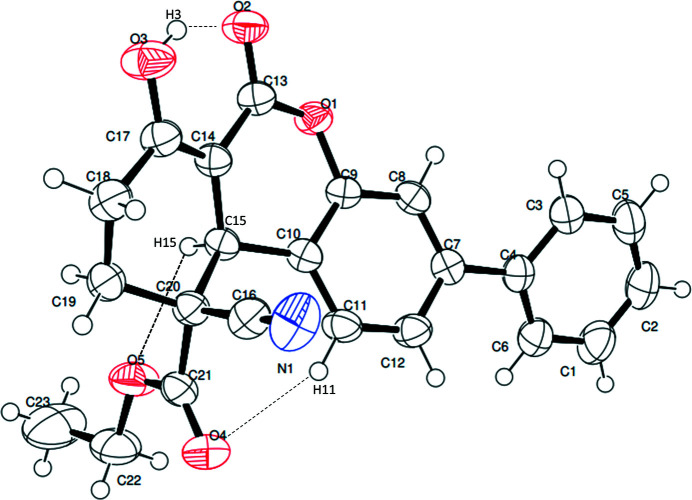
The mol­ecular structure of the title compound with the atom-numbering scheme and displacement ellipsoids drawn at the 50% probability level. Intra­molecular hydrogen bonds are shown as dashed lines.

**Figure 2:**
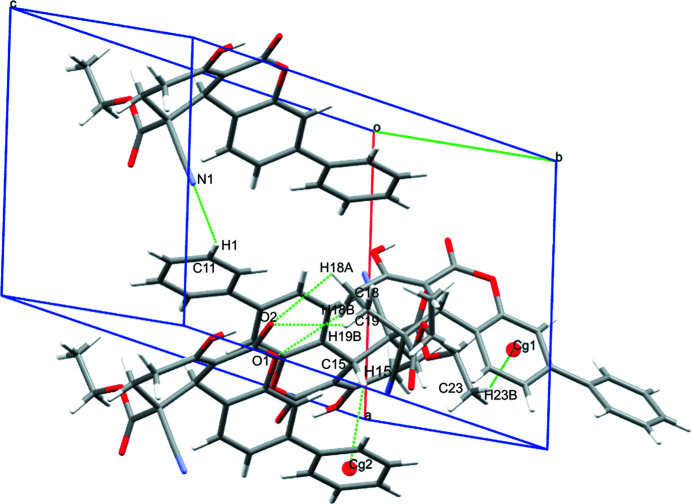
Inter­molecular inter­actions in the title compound.

The title compound has a dibenzo­pyran moiety decorated by several substituents, as shown in Fig. 1 ▸. There are two stereogenic centres: in the arbitrarily chosen asymmetric mol­ecule, C15 and C20 have *S* and *R* configurations, respectively, but crystal symmetry generates a racemic mixture. The nitrile group attached to C20 occupies an axial position and is *anti* to the hydrogen atom attached to C19. The dihedral angle between the pendant C1–C6 phenyl group and the C7–C12 benzene ring of the fused-ring system is 25.97 (8)°. The Cremer–Pople puckering parameters of the O1/C9/C10/C13–C15 and C14/C15/C17–C20 rings indicate half-chair conformations in each case with puckering amplitudes *Q* = 0.359 Å; θ = 104.52°; φ = 9.27° and *Q* = 0.49 Å; θ = 134.17°; φ = 327.35°, respectively. The O atom attached to C17 is stabilized in its enol (hy­droxy) form, presumably as a result of forming a strong intra­molecular hydrogen bond to O2. The packing is consolidated by weak C—H⋯O hydrogen bonds and C—H⋯π inter­actions (Table 1 ▸) and an intra­molecular C—H⋯O inter­action is also observed (Fig. 2 ▸).

From a Cambridge Structural Database search (Groom *et al.*, 2016 ▸), we found compounds identified by refcodes OKEYUB (Xiao *et al.*, 2021 ▸), QABVEY (Wang *et al.*, 2021 ▸), ALTENU (McPhail *et al.*, 1973 ▸), AMUYIS (Alzaydi *et al.*, 2016 ▸), ANOVEG (Sosnovskikh *et al.*, 2016 ▸), ANOVIK (Sosnovskikh *et al.*, 2016 ▸), BUWJEK (Parveen *et al.*, 2015 ▸), BUXLOW (Fatunsin *et al.*, 2010 ▸), DIPTUR (Casiraghi *et al.*, 1986 ▸), DISJAS (Lee *et al.*, 2013 ▸), SEDFEN (Appel *et al.*, 2006 ▸), SIVQIZ (Poudel & Lee, 2014 ▸), SIJZER (Hussain *et al.*, 2007 ▸), TUPJOE (Siegel *et al.*, 2010 ▸), ZAQHIK (Dasari *et al.*, 2012 ▸) and IZACIY (Duan *et al.*, 2021 ▸) to be similar to the title compound.

## Synthesis and crystallization

A mixture of ethyl 10-cyano-7-hy­droxy-6-oxo-3-{[(tri­fluoro­meth­yl)sulfon­yl]­oxy}-8,9,10,10*a*-tetra­hydro-6*H*-benzo[*c*]chromene-10-carboxyl­ate (100 mg, 0.22 mmol), phenyl­boronic acid (34 mg, 0.28 mmol, 1.3 equiv.), K_3_PO_4_ (73 mg, 0.34 mmol, 1.6 equiv.) and Pd(PPh_3_)_4_ (3 mg, 3 mol%) in degassed 1,4-dioxane (10 mL) was stirred at 100° C for 12 h under nitro­gen. After completion of the coupling reaction (TLC), the mixture was cooled to room temperature, diluted with di­chloro­methane (DCM, 10 mL) and deca­nted. The residue was extracted with DCM (10 mL × 2) twice. The solvent was removed from the combined DCM layers and the residue was subjected to column chromatography on silica gel (100–200 mesh) by using increasing amounts of ethyl acetate in hexane (5% to 15%) as eluent to afford the title compound as a light-yellow solid in 90% yield (84 mg); *R*_f_ = 0.4 (hexa­nes:ethyl acetate, 7:3); m.p. 155–158° C. A sample suitable for single-crystal X-ray analysis was obtained by recrystallization the 50 mg of the solid from a mixture of 1 mL of distilled chloro­form and 0.5 mL of distilled methanol.

## Refinement

Crystal data, data collection and structure refinement details are summarized in Table 2 ▸.

## Supplementary Material

Crystal structure: contains datablock(s) I. DOI: 10.1107/S2414314622001997/hb4401sup1.cif

Structure factors: contains datablock(s) I. DOI: 10.1107/S2414314622001997/hb4401Isup2.hkl

Supporting information file. DOI: 10.1107/S2414314622001997/hb4401Isup3.cml

CCDC reference: 2153368

Additional supporting information:  crystallographic information; 3D view; checkCIF report

## Figures and Tables

**Table 1:** Hydrogen-bond geometry (Å, °) *Cg*2 and *Cg*3 are the centroids of the C1–C6 and C7–C12 rings, respectively.

*D*—H⋯*A*	*D*—H	H⋯*A*	*D*⋯*A*	*D*—H⋯*A*
O3—H3⋯O2	0.82	1.86	2.5702 (16)	145
C11—H11⋯O4	0.93	2.54	3.399 (2)	154
C18—H18*B*⋯O1^i^	0.97	2.60	3.4289 (19)	144
C19—H19*B*⋯O2^i^	0.97	2.60	3.285 (2)	128
C15—H15⋯*Cg*2^ii^	0.98	2.95	3.7685 (17)	142
C23—H23*B*⋯*Cg*3^iii^	0.96	2.82	3.686 (3)	151

Symmetry codes: (i) 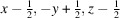 ; (ii) 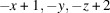 ; (iii) 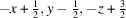 .

**Table 2:** Experimental details

Crystal data	
Chemical formula	C_23_H_19_NO_5_
*M*_r_	389.39
Crystal system, space group	Monoclinic, *P*2_1_/*n*
Temperature (K)	293
*a*, *b*, *c* (Å)	9.7089 (8), 14.3510 (12), 14.2749 (15)
β (°)	106.946 (10)
*V* (Å^3^)	1902.6 (3)
*Z*	4
Radiation type	Mo *K*α
μ (mm^−1^)	0.10
Crystal size (mm)	0.75 × 0.44 × 0.42

Data collection	
Diffractometer	Xcalibur, Eos
Absorption correction	Multi-scan (*CrysAlis PRO*; Agilent, 2014 ▸)
*T*_min_, *T*_max_	0.932, 1.000
No. of measured, independent and observed [*I* > 2σ(*I*)] reflections	10689, 4413, 3119
*R*_int_	0.026
(sin θ/λ)_max_ (Å^−1^)	0.686

Refinement	
*R*[*F* ^2^ > 2σ(*F* ^2^)], *wR*(*F* ^2^), *S*	0.046, 0.157, 0.95
No. of reflections	4413
No. of parameters	264
H-atom treatment	H-atom parameters constrained
Δρ_max_, Δρ_min_ (e Å^−3^)	0.19, −0.20

Computer programs: *CrysAlis PRO* (Agilent, 2014 ▸), *SHELXT* (Sheldrick, 2015*a* ▸), *SHELXL2018* (Sheldrick, 2015*b* ▸), *ORTEP-3 for Windows* (Farrugia, 2012 ▸), *PLATON* (Spek, 2020 ▸) and *Mercury* (Macrae *et al.*, 2020 ▸).

## full crystallographic data

### Crystal data

**Table:**

C_23_H_19_NO_5_	*F*(000) = 816
*M**_r_* = 389.39	*D*_x_ = 1.359 Mg m^−^^3^
Monoclinic, *P*2_1_/*n*	Mo *K*α radiation, λ = 0.71073 Å
*a* = 9.7089 (8) Å	Cell parameters from 2883 reflections
*b* = 14.3510 (12) Å	θ = 3.0–29.1°
*c* = 14.2749 (15) Å	µ = 0.10 mm^−^^1^
β = 106.946 (10)°	*T* = 293 K
*V* = 1902.6 (3) Å^3^	Block, colorless
*Z* = 4	0.75 × 0.44 × 0.42 mm

### Data collection

**Table:**

Xcalibur, Eos diffractometer	4413 independent reflections
Radiation source: Enhance (Mo) X-ray Source	3119 reflections with *I* > 2σ(*I*)
Detector resolution: 15.9821 pixels mm^-1^	*R*_int_ = 0.026
ω scans	θ_max_ = 29.2°, θ_min_ = 3.0°
Absorption correction: multi-scan (CrysalisPro; Agilent, 2014)	*h* = −13→13
*T*_min_ = 0.932, *T*_max_ = 1.000	*k* = −18→17
10689 measured reflections	*l* = −18→18

### Refinement

**Table:**

Refinement on *F*^2^	Primary atom site location: iterative
Least-squares matrix: full	Secondary atom site location: difference Fourier map
*R*[*F*^2^ > 2σ(*F*^2^)] = 0.046	Hydrogen site location: inferred from neighbouring sites
*wR*(*F*^2^) = 0.157	H-atom parameters constrained
*S* = 0.95	*w* = 1/[σ^2^(*F*_o_^2^) + (0.1*P*)^2^] where *P* = (*F*_o_^2^ + 2*F*_c_^2^)/3
4413 reflections	(Δ/σ)_max_ = 0.010
264 parameters	Δρ_max_ = 0.19 e Å^−^^3^
0 restraints	Δρ_min_ = −0.20 e Å^−^^3^

### Special details

**Table:**

Geometry. All esds (except the esd in the dihedral angle between two l.s. planes) are estimated using the full covariance matrix. The cell esds are taken into account individually in the estimation of esds in distances, angles and torsion angles; correlations between esds in cell parameters are only used when they are defined by crystal symmetry. An approximate (isotropic) treatment of cell esds is used for estimating esds involving l.s. planes.
Refinement. Refinement of F^2^ against ALL reflections. The weighted R-factor wR and goodness of fit S are based on F^2^, conventional R-factors R are based on F, with F set to zero for negative F^2^. The threshold expression of F^2^ > 2sigma(F^2^) is used only for calculating R-factors(gt) etc. and is not relevant to the choice of reflections for refinement. R-factors based on F^2^ are statistically about twice as large as those based on F, and R- factors based on ALL data will be even larger. The hydrogen atoms in title compound were placed in calculated positions, with C—H = 0.93–0.97 A° and refined using a riding model with *U*_iso_(H) = 1.2 *U_eq_*(C) or 1.5*U*_eq_(*C*-methyl).

### Fractional atomic coordinates and isotropic or equivalent isotropic displacement parameters (Å^2^)

**Table:**

	*x*	*y*	*z*	*U*_iso_*/*U*_eq_	
O1	0.53859 (10)	0.16733 (8)	0.92065 (7)	0.0410 (3)	
O2	0.63028 (12)	0.27465 (8)	0.84841 (8)	0.0509 (3)	
C14	0.44382 (14)	0.18379 (10)	0.74486 (11)	0.0347 (3)	
O5	0.27716 (13)	−0.06937 (8)	0.61784 (10)	0.0595 (4)	
C15	0.36436 (14)	0.09212 (10)	0.73953 (10)	0.0332 (3)	
H15	0.431514	0.042889	0.734017	0.040*	
O3	0.50815 (13)	0.31695 (9)	0.66833 (9)	0.0588 (4)	
H3	0.561932	0.325224	0.723781	0.088*	
C9	0.42029 (14)	0.11197 (10)	0.92053 (11)	0.0337 (3)	
C10	0.32907 (15)	0.07558 (10)	0.83485 (11)	0.0347 (3)	
C7	0.29134 (15)	0.04148 (11)	1.02203 (11)	0.0366 (4)	
C4	0.27309 (15)	0.02080 (11)	1.11980 (11)	0.0382 (4)	
C19	0.28311 (17)	0.11903 (13)	0.55664 (11)	0.0447 (4)	
H19A	0.360887	0.079214	0.550748	0.054*	
H19B	0.203779	0.112652	0.497179	0.054*	
C16	0.11816 (17)	0.14945 (12)	0.65498 (12)	0.0450 (4)	
C13	0.54193 (15)	0.21194 (11)	0.83729 (11)	0.0375 (4)	
C20	0.23367 (15)	0.08697 (11)	0.64512 (11)	0.0377 (4)	
C8	0.40422 (15)	0.09584 (10)	1.01183 (11)	0.0366 (4)	
H8	0.469369	0.121527	1.066950	0.044*	
C21	0.17320 (17)	−0.01170 (12)	0.62229 (11)	0.0437 (4)	
O4	0.05006 (13)	−0.03278 (10)	0.60776 (10)	0.0649 (4)	
C12	0.19570 (17)	0.00624 (12)	0.93635 (12)	0.0443 (4)	
H12	0.117331	−0.029036	0.940506	0.053*	
C18	0.33369 (17)	0.21905 (12)	0.56740 (12)	0.0467 (4)	
H18A	0.381800	0.232559	0.518170	0.056*	
H18B	0.250752	0.259856	0.555766	0.056*	
C17	0.43402 (16)	0.23902 (11)	0.66616 (12)	0.0410 (4)	
C11	0.21487 (17)	0.02259 (12)	0.84563 (12)	0.0443 (4)	
H11	0.149569	−0.002549	0.790253	0.053*	
C6	0.20395 (18)	−0.05906 (13)	1.13558 (13)	0.0507 (4)	
H6	0.166517	−0.099657	1.083600	0.061*	
C3	0.32765 (18)	0.08032 (12)	1.19927 (12)	0.0466 (4)	
H3A	0.375026	0.134713	1.191218	0.056*	
C2	0.2424 (2)	−0.02057 (16)	1.30377 (15)	0.0627 (5)	
H2	0.231601	−0.034154	1.364935	0.075*	
C1	0.1887 (2)	−0.08045 (15)	1.22642 (15)	0.0637 (5)	
H1	0.142442	−0.135050	1.235243	0.076*	
C5	0.3116 (2)	0.05875 (15)	1.29030 (14)	0.0579 (5)	
H5	0.348412	0.098916	1.342756	0.070*	
C22	0.2376 (2)	−0.16616 (14)	0.59282 (18)	0.0714 (6)	
H22A	0.150569	−0.169084	0.538150	0.086*	
H22B	0.220030	−0.197877	0.648226	0.086*	
C23	0.3565 (3)	−0.21069 (18)	0.5666 (2)	0.1099 (10)	
H23A	0.372605	−0.179133	0.511451	0.165*	
H23B	0.333040	−0.274743	0.550071	0.165*	
H23C	0.442123	−0.207456	0.621170	0.165*	
N1	0.03398 (17)	0.20167 (13)	0.66178 (13)	0.0658 (5)	

### Atomic displacement parameters (Å^2^)

**Table:**

	*U*^11^	*U*^22^	*U*^33^	*U*^12^	*U*^13^	*U*^23^
O1	0.0360 (5)	0.0501 (7)	0.0321 (6)	−0.0093 (5)	0.0023 (4)	0.0025 (5)
O2	0.0501 (7)	0.0508 (7)	0.0448 (7)	−0.0184 (6)	0.0029 (5)	0.0017 (5)
C14	0.0322 (7)	0.0351 (8)	0.0334 (8)	−0.0002 (6)	0.0042 (6)	0.0018 (6)
O5	0.0520 (7)	0.0435 (7)	0.0799 (10)	−0.0124 (6)	0.0143 (6)	−0.0140 (6)
C15	0.0313 (7)	0.0349 (8)	0.0296 (8)	0.0014 (6)	0.0027 (6)	0.0016 (6)
O3	0.0606 (8)	0.0537 (8)	0.0516 (8)	−0.0181 (6)	−0.0003 (6)	0.0162 (6)
C9	0.0306 (7)	0.0323 (8)	0.0347 (8)	0.0019 (6)	0.0041 (6)	0.0024 (6)
C10	0.0364 (7)	0.0323 (8)	0.0318 (8)	0.0017 (6)	0.0040 (6)	0.0029 (6)
C7	0.0385 (7)	0.0357 (8)	0.0338 (9)	0.0056 (6)	0.0078 (6)	0.0026 (6)
C4	0.0365 (7)	0.0410 (9)	0.0375 (9)	0.0092 (6)	0.0115 (6)	0.0034 (7)
C19	0.0431 (8)	0.0569 (11)	0.0311 (9)	−0.0059 (8)	0.0060 (6)	0.0023 (7)
C16	0.0375 (8)	0.0543 (10)	0.0396 (10)	0.0015 (8)	0.0056 (6)	0.0104 (8)
C13	0.0334 (7)	0.0373 (8)	0.0383 (9)	−0.0003 (6)	0.0051 (6)	0.0039 (7)
C20	0.0341 (7)	0.0448 (9)	0.0307 (8)	−0.0029 (6)	0.0039 (6)	0.0012 (7)
C8	0.0379 (7)	0.0349 (8)	0.0324 (8)	0.0026 (6)	0.0028 (6)	−0.0005 (6)
C21	0.0431 (9)	0.0534 (10)	0.0307 (9)	−0.0102 (8)	0.0049 (6)	−0.0038 (7)
O4	0.0483 (7)	0.0789 (10)	0.0652 (9)	−0.0248 (7)	0.0132 (6)	−0.0154 (7)
C12	0.0437 (8)	0.0481 (10)	0.0389 (9)	−0.0103 (7)	0.0086 (7)	0.0044 (7)
C18	0.0430 (9)	0.0564 (11)	0.0361 (9)	−0.0032 (7)	0.0045 (7)	0.0121 (8)
C17	0.0377 (8)	0.0423 (9)	0.0399 (9)	−0.0027 (7)	0.0064 (6)	0.0052 (7)
C11	0.0444 (8)	0.0481 (10)	0.0344 (9)	−0.0110 (7)	0.0022 (7)	0.0010 (7)
C6	0.0548 (10)	0.0529 (11)	0.0485 (11)	−0.0025 (8)	0.0214 (8)	0.0004 (8)
C3	0.0476 (9)	0.0504 (10)	0.0426 (10)	0.0034 (8)	0.0144 (7)	−0.0021 (8)
C2	0.0683 (12)	0.0819 (15)	0.0468 (12)	0.0071 (11)	0.0307 (10)	0.0081 (10)
C1	0.0711 (12)	0.0699 (14)	0.0602 (13)	−0.0059 (11)	0.0348 (10)	0.0087 (10)
C5	0.0591 (11)	0.0746 (13)	0.0424 (11)	0.0072 (10)	0.0185 (8)	−0.0082 (9)
C22	0.0747 (13)	0.0481 (12)	0.0924 (17)	−0.0228 (10)	0.0258 (11)	−0.0196 (11)
C23	0.100 (2)	0.0564 (15)	0.188 (3)	−0.0203 (13)	0.065 (2)	−0.0405 (17)
N1	0.0558 (9)	0.0742 (11)	0.0700 (12)	0.0198 (9)	0.0222 (8)	0.0222 (9)

### Geometric parameters (Å, º)

**Table:**

O1—C13	1.3599 (18)	C7—C4	1.487 (2)
O1—C9	1.3961 (17)	C4—C6	1.379 (2)
O2—C13	1.2212 (18)	C4—C3	1.395 (2)
C14—C17	1.355 (2)	C19—C18	1.510 (2)
C14—C13	1.442 (2)	C19—C20	1.546 (2)
C14—C15	1.516 (2)	C16—N1	1.134 (2)
O5—C21	1.321 (2)	C16—C20	1.474 (2)
O5—C22	1.457 (2)	C20—C21	1.531 (2)
C15—C10	1.516 (2)	C21—O4	1.1913 (18)
C15—C20	1.5604 (19)	C12—C11	1.381 (2)
O3—C17	1.3255 (19)	C18—C17	1.489 (2)
C9—C8	1.377 (2)	C6—C1	1.382 (2)
C9—C10	1.386 (2)	C3—C5	1.388 (2)
C10—C11	1.390 (2)	C2—C5	1.363 (3)
C7—C8	1.387 (2)	C2—C1	1.376 (3)
C7—C12	1.397 (2)	C22—C23	1.460 (3)
			
C13—O1—C9	119.76 (11)	O1—C13—C14	119.40 (13)
C17—C14—C13	117.57 (14)	C16—C20—C21	109.13 (13)
C17—C14—C15	123.65 (13)	C16—C20—C19	108.83 (12)
C13—C14—C15	118.68 (13)	C21—C20—C19	107.00 (13)
C21—O5—C22	117.30 (14)	C16—C20—C15	109.78 (13)
C14—C15—C10	109.62 (12)	C21—C20—C15	113.11 (12)
C14—C15—C20	111.00 (12)	C19—C20—C15	108.88 (12)
C10—C15—C20	115.36 (12)	C9—C8—C7	120.33 (13)
C8—C9—C10	123.53 (14)	O4—C21—O5	125.08 (16)
C8—C9—O1	114.50 (12)	O4—C21—C20	125.09 (16)
C10—C9—O1	121.96 (13)	O5—C21—C20	109.79 (13)
C9—C10—C11	115.70 (14)	C11—C12—C7	121.50 (15)
C9—C10—C15	118.66 (13)	C17—C18—C19	112.50 (13)
C11—C10—C15	125.57 (13)	O3—C17—C14	124.59 (14)
C8—C7—C12	117.11 (14)	O3—C17—C18	112.66 (13)
C8—C7—C4	121.65 (13)	C14—C17—C18	122.73 (14)
C12—C7—C4	121.25 (14)	C12—C11—C10	121.79 (14)
C6—C4—C3	117.61 (15)	C4—C6—C1	121.87 (18)
C6—C4—C7	121.05 (15)	C5—C3—C4	120.32 (17)
C3—C4—C7	121.33 (15)	C5—C2—C1	119.68 (18)
C18—C19—C20	111.65 (14)	C2—C1—C6	119.68 (19)
N1—C16—C20	176.08 (18)	C2—C5—C3	120.84 (18)
O2—C13—O1	115.36 (13)	O5—C22—C23	107.97 (17)
O2—C13—C14	125.24 (14)		

### Hydrogen-bond geometry (Å, º)

*Cg*2 and *Cg*3 are the centroids of the C1–C6 and C7–C12 rings, respectively.

**Table:**

*D*—H···*A*	*D*—H	H···*A*	*D*···*A*	*D*—H···*A*
O3—H3···O2	0.82	1.86	2.5702 (16)	145
C11—H11···O4	0.93	2.54	3.399 (2)	154
C18—H18*B*···O1^i^	0.97	2.60	3.4289 (19)	144
C19—H19*B*···O2^i^	0.97	2.60	3.285 (2)	128
C15—H15···*Cg*2^ii^	0.98	2.95	3.7685 (17)	142
C23—H23*B*···*Cg*3^iii^	0.96	2.82	3.686 (3)	151

Symmetry codes: (i) *x*−1/2, −*y*+1/2, *z*−1/2; (ii) −*x*+1, −*y*, −*z*+2; (iii) −*x*+1/2, *y*−1/2, −*z*+3/2.
